# Estimation of the Onset of Crack Growth in Ductile Materials

**DOI:** 10.3390/ma11102026

**Published:** 2018-10-18

**Authors:** Andrzej Neimitz, Jaroslaw Galkiewicz, Sebastian Lipiec, Ihor Dzioba

**Affiliations:** Faculty of Mechatronics and Mechanical Engineering, Kielce University of Technology, Aleja Tysiaclecia Panstwa Polskiego 7, 25-314 Kielce, Poland; jgalka@tu.kielce.pl (J.G.); slipiec@tu.kielce.pl (S.L.); pkmid@tu.kielce.pl (I.D.)

**Keywords:** ductile fracture, ductile fracture mechanisms, critical effective plastic strain, stress triaxiality, Lode angle

## Abstract

In this paper, the ductile fracture mechanism is discussed. The results of numerical and experimental analyses were used to estimate the onset of crack front growth. It was assumed that the ductile fracture in front of the crack starts at the location along the crack front where the accumulated effective plastic strain reaches a critical value. According to numerous research articles, the critical effective plastic strain depends on the stress triaxiality and the Lode angle. The experimental program was performed using five different specimen geometries, three different materials, and three different temperatures of +20 °C, −20 °C, and −50 °C. Using the experimental data and results of the finite element computations, the critical effective plastic strains were determined for each material and temperature. However, before the critical effective plastic strain was determined, a careful calibration of the stress–strain curves was performed after modification of the Bai–Wierzbicki procedure. It was found that critical effective plastic strain was a function of triaxiality factor and Lode parameter, as expected, and that the fracture locus was useful to estimate the onset of ductile crack growth.

## 1. Introduction

**Figure 1:**
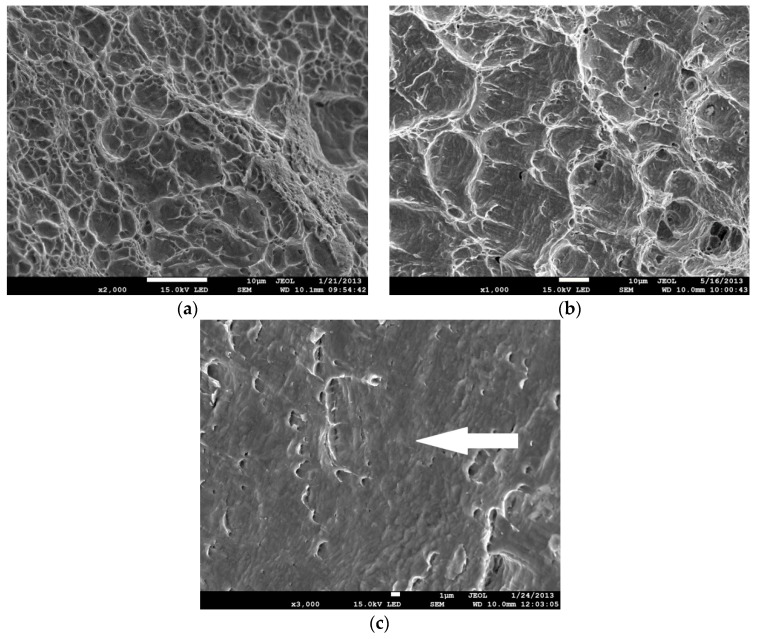
The influence of stress triaxiality on the ductile fracture mechanism. Material Hardox-400 (Source: own research). (**a**) Dimples in front of the crack, central part of the specimen (*η* = 2.5). (**b**) Dimples in front of the crack, located at the mid-part of the shear lips. Parabolic shape of the dimples is due to the shear stress (*η* = 1.6). (**c**) The traces of dimples in front of the crack, located close to the specimen surface–shear lips (*η* = 0.9). Arrow points to the shear planes and failure domain due to the dislocations slip.

The failure of ferritic steels covers a wide spectrum of fracture mechanisms that depend on both microstructure and temperature. At room temperature or at temperatures that are not too low, ductile fracture dominates. However, although the ductile fracture mechanism, in most cases, is a result of voids nucleation, growth, and coalescence, different images of fracture surfaces can be observed. The images are a result of different levels of stress triaxialities and Lode angles (factors) [1,2]. When the stress triaxiality is high, the dimples are deep, and the coalescence mechanism is caused by necking of intervoid ligaments (Figure 1a). When the stress triaxiality is low, the dimples are shallow and elongated, suggesting significant shear plastic strains and shear localization between voids (Figure 1b). In addition, a ductile failure may take place as a result of a dislocation’s glide along the slip planes (Figure 1c).

Here, the stress triaxiality is measured using the *η* parameter:

(1) $$\eta =\frac{\sigma_{m}}{\sigma_{e}}\;$$

where *σ_m_* and *σ_e_* are the first stress tensor invariant and effective stress, respectively; $\sigma_{e}=\sqrt{3J_{2}}$ and *J*_2_ is the second stress deviator invariant.

All these mechanisms of ductile fracture can be observed in front of the crack. However, the stress triaxiality factor alone is not always sufficient to explain changes in the failure mechanism. It has been suggested that other parameters that could be helpful in qualitative and quantitative analysis of fracture mechanisms are the accumulated effective plastic strain and the Lode angle/factor [3]. The Lode angle *θ*, or one of the Lode parameters *ξ*, or *L* are defined as follows:

(2) $$\cos\left(3\theta \right)={\left(r/\sigma_{e}\right)}^{3}=\xi =27/2\cdot J_{3}/\sigma_{e}^{3}\;$$

(3) $$r={\left[27/2\det\left(s_{ij}\right)\right]}^{1/3}={\left[27/2\left(\sigma_{1}-\sigma_{m}\right)\left(\sigma_{2}-\sigma_{m}\right)\left(\sigma_{3}-\sigma_{m}\right)\right]}^{1/3}\;$$

where *θ* is the Lode angle; *s_ij_* is the stress tensor deviator; and *J*_3_ is the third invariant of the stress deviator. In this paper, the Lode factor was used, the value of which can be computed from the following formula:

(4) $$L=-\frac{2\sigma_{II}-\sigma_{I}-\sigma_{III}}{\sigma_{I}-\sigma_{III}}\;$$

and *L* is related to the *ξ* function by the relationship:

(5) $$\xi =L\left(9-L^{2}\right)/\sqrt{{\left(L^{2}+3\right)}^{3}}\;$$

In Equation (4), *σ_I_* is the highest principal stress and *σ_III_* is the smallest principal stress; *L* = 1 (or *L* = −1) [4] when the axial symmetric tensile (or compressive) loading is met; *L* = 0 when pure shear is met in the plane stress state. Other loading cases are located in between the above values.

**Figure 2:**
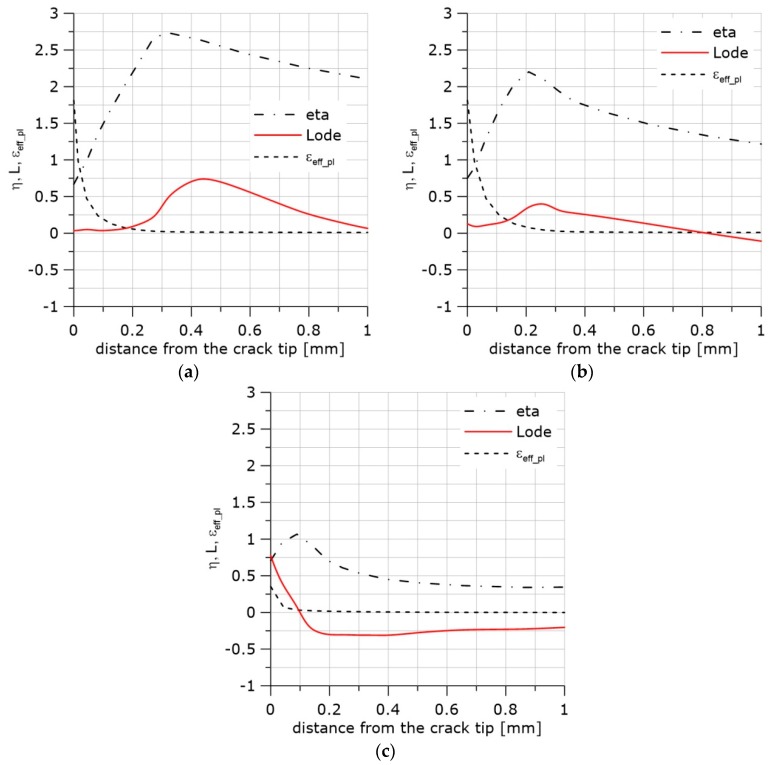
Distributions of *η*, *L*, and *ε_eff_pl_* in front of the crack. Material S355JR (heat treatment NW, Table 1). (**a**) The central part of the specimen; (**b**) the layer located 1.75 mm from the surface; (**c**) the next to last layer from the specimen axis.

The exemplary distributions of *η*, *L*, and the accumulated effective plastic strain, *ε_eff_pl_*, along the crack front are shown in Figure 2.

The curves in Figure 2 were obtained by the finite element method using the ABAQUS program (Abaqus/CAE 6.12-2, Dassault Systemes Simulia Corp, Providence, RI, USA). The boundary conditions were adopted from the experiment with the single edge notch bend (SEN(B)) specimen. The thickness of the specimen was B = 12 mm, and the width was W = 24 mm. Half of the specimen thickness was divided into 10 layers. The contact problem was considered. The displacement of the loading pin was taken from the experiment in the cases shown in Figure 2 and other SEN(B) specimens tested in this research.

Large strains and *J*_2_ plasticity were assumed. Linear, hexagonal C3D8 elements (ABAQUS) with full integration were used. The crack tip was blunted by a 10 μm radius. The size of the finite elements increased with the distance from the crack tip. The size of the smallest element in the radial direction was 27 μm. The thickness of the layers decreased towards the specimen’s external surface; the thinnest layer was 0.27 mm.

In contrast to the cleavage fracture, it is very difficult to predict the onset of crack growth in ductile materials. Ductile crack growth, due to voids nucleation–growth–coalescence, is found to initiate at different moments at different locations along the blunted crack front. This moment is not noticeable when the load–deflection curve is observed, and the onset of a crack growth usually occurs when this curve is still rising. This trait is a reason why standards are proposed to measure the critical value of *J*-integral after the presumable 0.2 mm average crack front extension.

The purpose of the research program reported in this paper was to estimate and predict the moment and location of the onset of the ductile crack extension.

The research hypothesis was that a crack starts growing by the void nucleation–growth–coalescence mechanism when the accumulated effective plastic strain reaches the critical value (*ε_eff_pl_* = *ε_eff_pl_cr_*). Following the results of Bao, Bai, and Wierzbicki [1,3,4,5,6,7] among others [8,9,10,11], it was assumed that for both steel [12,13] and aluminum [14], the critical effective plastic strain depends on the triaxiality parameter and the Lode angle/factor [13,15,16].

## 2. Materials and Tested Specimens

**Figure 3:**
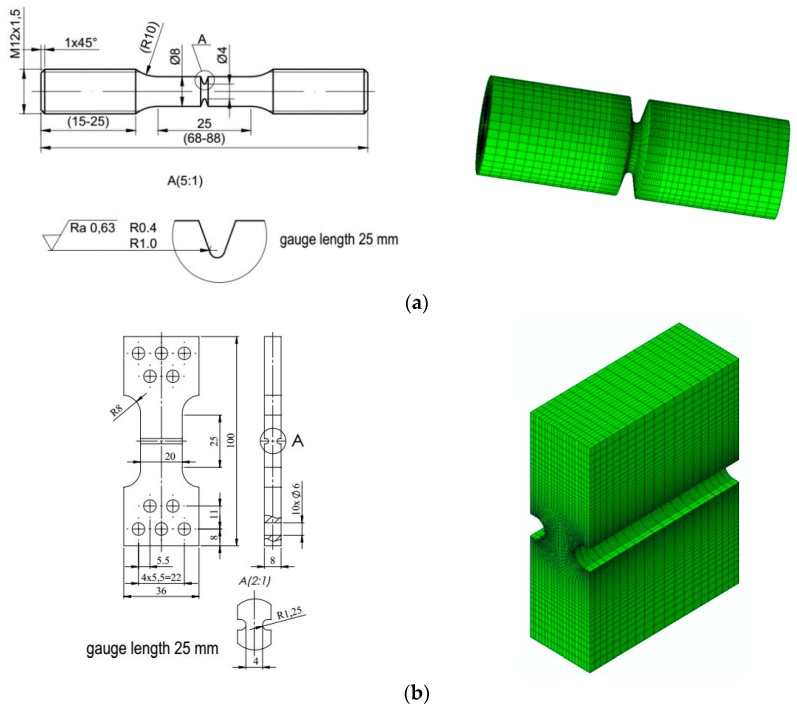

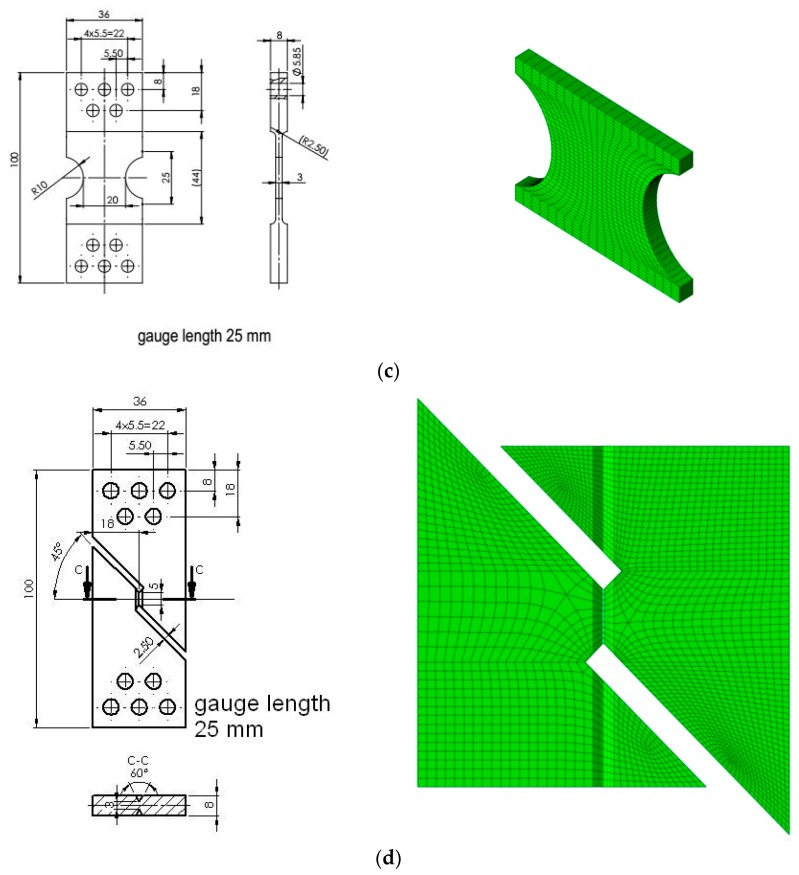
Mechanical drawings and finite element mesh images of tested specimens: (**a**) two-notched cylindrical specimens C04 with *R* = 0.4 mm (*η* ≈ from 0.5 to 1.6, *L* ≈ from 0.6 to 1) and C1 with *R* = 1 mm (*η* ≈ from 0.4 to 1.4, *L* ≈ from 0.85 to 1); (**b**) plate with side groove (PN), *R* = 1 mm (*η* ≈ 0.4, *L* = 0.4); (**c**) plate with *R* = 10 mm (PR) (*η* ≈ 0.5, *L* = 0.5); and (**d**) pure shear (S) (*η* ≈ 0, *L* = 0) (Source: authors’ drawings).

The calibration of the constitutive equations and determination of the critical values of the accumulated effective plastic strains were performed using the specimens shown in Figure 3.

The mechanical properties of the S355JR (1.0045) steel under different heat treatments tested in the research programs are listed in Table 1. Another symbol of this steel is 18G2A according to Polish standards, and A678Gr.A according to USA standards. It is a nonalloy quality structural steel widely applied for welded structures, bridges, reinforced concrete bars, and structures working at low temperatures. Heat treatment was performed to obtain different shapes and sizes of carbides from pearlite to spheroidal shapes. Different temperature levels were used to control the extent of plasticity.

## 3. Calibration of the Constitutive Relationships

To perform finite element analysis on any shape and size of a machine or structural member, uniaxial stress–strain curves are required; results of standard uniaxial tensile tests alone are not sufficient, especially when large plastic strains are expected. It is not sufficient to only convert stresses to the true stresses and strains to the logarithmic strains; the stress triaxiality and the way a specimen is loaded should also be taken into account to ensure conformity between numerical and experimental results. Thus, the stress–strain curves must be calibrated before they are used by the finite element code. In research papers where both experimental and numerical results are used to prove some hypotheses, the stress–strain relationship problem is often not discussed. The calibration of tensile test curves is either not performed or is a result of curve fitting by trial and error. However, it is sometimes performed by a well-defined methodology [3,4,13]. In this research program, the Bai–Wierzbicki (BW) methodology [3] was adopted, which involves both the stress triaxiality factor *η* and the Lode parameter. Equation (6) was selected from several equivalent formulae proposed by BW and other authors:

(6) $$\sigma_{yld}=\bar{\sigma }\left({\bar{\varepsilon }}_{p}\right)\left[1-c_{\eta }\left(\eta -\eta_{0}\right)\right]\left[c_{\theta }^{s}+\left(c_{\theta }^{ax}-c_{\theta }^{s}\right)\left(\gamma -\frac{\gamma^{m+1}}{m+1}\right)\right]\;$$

where *η*_0_ is a reference value of the triaxiality coefficient and *η_0_* = 1/3 for the uniaxial tensile test. The *γ* function represents a curve drawn at the deviatoric surface between the contours defined by the Huber–von Mises and Tresca criteria in the principal stress space. The *γ* function satisfies the inequality 0 ≤ *γ* ≤ 1, where *γ* = 0 for plane stress or pure shear, and *γ* = 1 for axial symmetry. BW postulated that the *γ* function takes the following form:

(7) $$\gamma =\frac{\cos\left(\pi /6\right)}{1-\cos\left(\pi /6\right)}\left[\frac{1}{\cos\left(\theta -\pi /6\right)}-1\right]=6.464\left[\sec\left(\theta -\pi /6\right)-1\right]\;$$

In Equation (6), the quantity $c_{\theta }^{ax}$ is defined as follows:

(8) $$c_{\theta }^{ax}=\begin{array}{cc} c_{\theta }^{t} & for\;\bar{\theta }\geq 0\\ c_{\theta }^{c} & for\;\bar{\theta }<0 \end{array}\;$$

$\bar{\theta }=1-6\theta /\pi$. Equation (6) contains four parameters to be determined: $c_{\theta }^{t}$, $c_{\theta }^{c}$, $c_{\theta }^{s}$, and *m*. The term containing the *m* parameter is added to make the yield surface smooth and differentiable with respect to the Lode angle *θ* in the neighborhood of *γ* = 1. These parameters must be determined experimentally; however, at least one of them is equal to unity. If $\bar{\sigma }\left({\bar{\varepsilon }}_{p}\right)$ is found through a uniaxial tensile test using cylindrical specimens, then $c_{\theta }^{t}=1$. If a uniaxial compression test is performed, then $c_{\theta }^{c}=1$, and in the case of a shear test, $c_{\theta }^{s}=1$. In the original BW methodology, both *η* and *θ* parameters are assumed constant during the specimen loading. Here, it was assumed that both *η* and *L* parameters change over the critical plane and over time during the loading process. The average values of these quantities over the critical plane were introduced into Equation (6), and the *η* function therefore changed according to Equation (9):

(9) $$\eta =\eta_{i}-\left(\frac{\eta_{i}-\eta_{f}}{\varepsilon_{pl\_avr\_final}}\right)\varepsilon_{pl\_avr}\;$$

where index *i* denotes the initial state, index *f* denotes the final state; *ε_pl_avr_final_* is the average value of the effective plastic strain in the critical plane before the failure; and *ε_pl_avr_* is the actual average effective plastic strain in the critical plane. A similar formula was used for the Lode parameter. The average values over the critical plane were assumed because the force–elongation curve represents the average response of the specimen to external loading.

Comparison of the experimental and numerical results during the calibration process led to the conclusion that softening of the material during the loading process caused by the void growth and coalescence at the final stage of loading should also be taken into account. Thus, it was assumed that *c_η_* in Equation (6) took the following form:

(10) $$c_{\eta }=\alpha {\left[1+H\left(\varepsilon_{eff\_pl}-\varepsilon_{eff\_pl\_o}\right)\left(\varepsilon_{eff\_pl}-\varepsilon_{eff\_pl\_o}\right)\right]}^{\zeta }\;$$

where *ε_eff_pl_o_* denotes the value of the effective plastic strain at the presumed onset of rapid void growth; H(*ε_eff_pl_–ε_eff_pl_o_*) is the Heaviside step function; coefficients *α* and *ζ* should be determined experimentally.

More details concerning the calibration of the stress–strain curves according to the procedure described above can be found in Reference [17].

**Figure 4:**
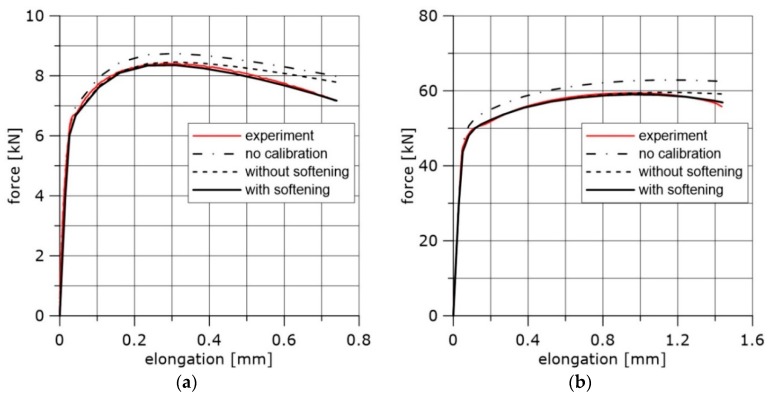

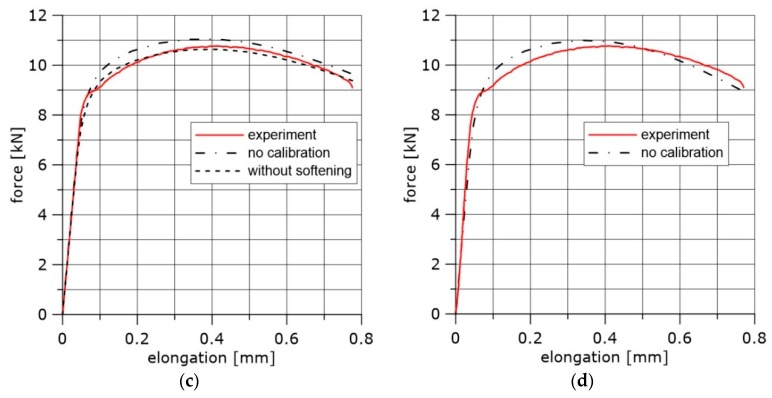
The force–elongation curves obtained before and after calibration of the true stress–logarithmic strain curves, (**a**) Material NW, temperature +20 °C, specimen used for calibration: R1. (**b**) Material HW, temperature +20 °C, specimen used for calibration: PN. (**c**) Material N, temperature −50 °C, specimen used for calibration: R04, linear approximation. (**d**) Material N, temperature −50 °C, specimen used for calibration: R04, power function approximation.

In Figure 4, the exemplary curves determined experimentally and numerically after and before calibration are presented. The examples cover the whole temperature range and three specimens.

Before calibration, each stress–strain curve was converted to the true stress–logarithmic strain (TS–LS) curve; after the maximum was reached, it was extrapolated either as a power function or linear function. An approximation of the TS–LS curve by a power function leads to good results for plastic materials at room temperature. However, when the test temperature is lowered, such an approximation is not always recommended because, in many cases, using the power function may lead to a situation where the calibration procedure proposed in this paper cannot be applied, see Figure 4d.

## 4. Critical Accumulated Effective Plastic Strain

According to several research reports, beginning with studies by McCintock [18] and Rice and Tracey [19], the critical strain at the onset of ductile failure depends (at least) on stress triaxiality measured, for example, by the *η* parameter. Recently, it was noticed [1,2] that the critical strain also depends on the Lode angle or Lode parameter defined in Section 1. The Lode angle describes how the specimen or structural member is loaded.

The specimens used for the TS–LS curves calibration were also used for estimation of the critical effective plastic strains for all three materials and three temperatures.

**Figure 5:**
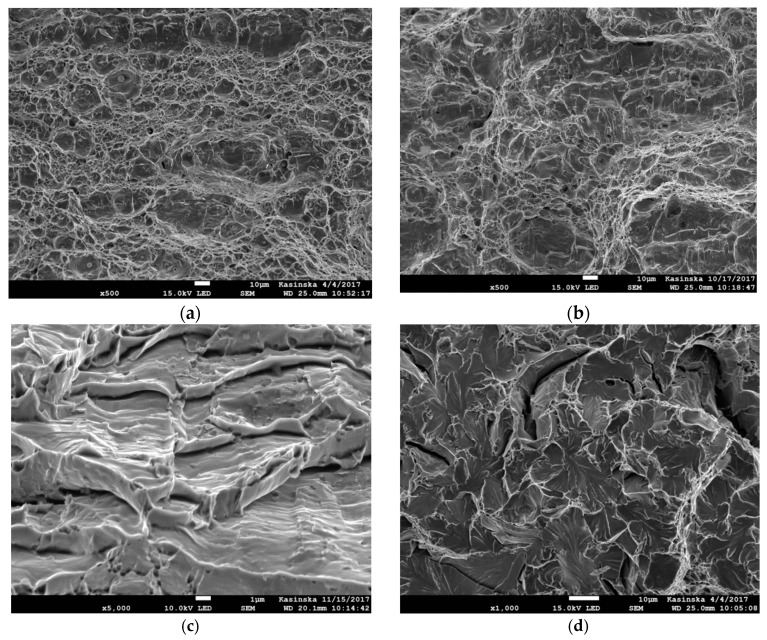
Images of the fracture surfaces of different specimen shapes. (**a**) Dimples along the fracture surface in specimen C1, material N, temperature +20 °C. (**b**) Dimples along the fracture surface in specimen PN, material N, temperature +20 °C. (**c**) Ductile fracture due to the dislocations glides along the slip planes; specimen PR, material HW, temperature −20 °C. (**d**) Cleavage fracture next to the circumferential notch in specimen C04, material NW, temperature −50 °C.

After specimen failure, the fracture surfaces of broken specimens were observed using electron scanning microscope, and finite element analyses were performed using the ABAQUS program. During numerical computations, the finite elements from the ABAQUS standard library were used. In the case of specimens C04 and C1, 4-node, reduced-integration, axisymmetric, solid elements were used (symbol CAX4R). Because large gradients of the computed quantities were not expected, the size of the element next to the notch was 0.138 mm. The other two cases (PN and PR specimens) were modeled using linear 3D hexagonal elements with reduced integration (C3D8R). The sizes of the element in the direction of the greatest stress gradient were 1/20 width of the specimen, i.e., 1.0 mm for the PR specimen and 0.086 mm for the PN specimen. In the case of the S specimen, the C3D8R elements were used, and the size of the element in the shear region was 0.2 mm. The symmetries of the modeled specimens were taken into account to reduce the time of computations. The specimens were loaded by displacements applied at the distance determined by the gauge length (see Figure 3). As a result of numerical computations, the following quantities were recorded over the critical plane: *η*, *L*, *ε_eff_pl_cr_*, and *σ*_22_, where *σ*_22_ is the crack faces opening stress. In most cases, the microscopic observations revealed the ductile failure via the void mechanism (Figure 5a,b); in some cases, failure due to the slip over slip planes (Figure 5c) took place (in this case, the *L* parameter must be close to zero), and in some cases, the cleavage failure mechanism (Figure 5d) was observed. The latter mechanism was observed at low temperatures.

In most cases, microscopic observations alone are not sufficient to decide at which part of the critical plane the final stage of the void growth–coalescence process initiated. It was assumed that at such a place, the dimples must be the largest. In most cases, both within cylindrical and PN specimens, the differences between the sizes and shapes of caverns were not clearly noticeable. Thus, a working hypothesis had to be assumed to localize the critical spot. The origin of this hypothesis was Rice and Tracy’s [19] results concerning the rate of growth of the isolated spherical void surrounded by an ideally plastic material. Their numerical results were well approximated by the following formula:$\frac{{\dot{R}}_{0}}{R_{0}}≅0.283\dot{\varepsilon }\exp\left(\frac{3\sigma_{m}}{2\sigma_{e}}\right)$. Because the whole critical cross section of the loaded specimen was stretched at the same time, it was proposed to compare the quantity representing, in a very rough approximation, the extension of the voids’ radii, which was recorded along the fractured surface at the presumed moment of the rapid evolution of damage. The simplified formula is as follows:

(11) $$\Delta R=\Delta \varepsilon_{eff\_pl}\exp\left(\eta \right)\;$$

**Figure 6:**
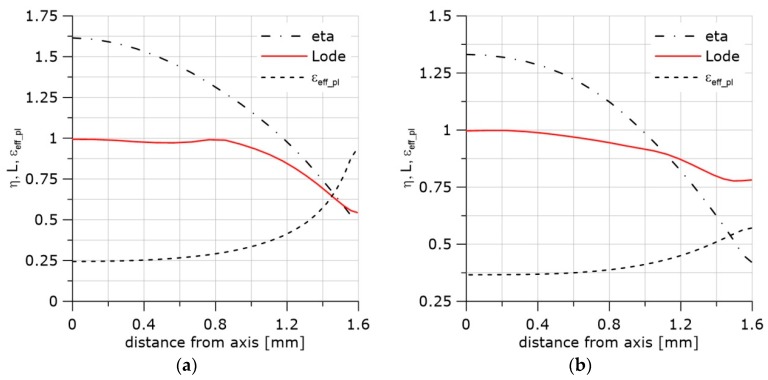
Distribution of *η*, *L*, *ε_eff_pl_* functions. (**a**) Specimen C04, material HW, temperature +20 °C, last step of the integration. (**b**) Specimen C1, material HW, temperature +20 °C, last step of the integration.

The exemplary results concerning two cylindrical specimens with different radii at the bottom of the circumferential notch are shown in Figure 6 and Table 2.

**Figure 7:**
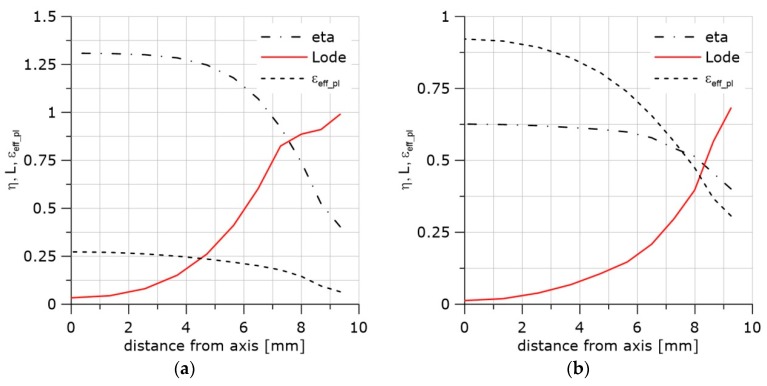

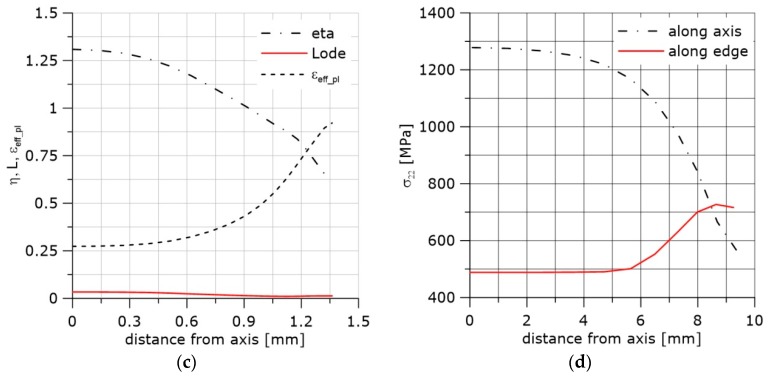
Distribution of *η*, *L*, *ε_eff_pl_* functions. (**a**) Curves drawn along the longer specimen axis, specimen PN, material HW, temperature +20 °C, last step of the integration. (**b**) Curves drawn along the notch, specimen PN, material HW, temperature +20 °C, last step of the integration. (**c**) Curves drawn along the shorter specimen axis, specimen PN, material HW, temperature +20 °C, last step of the integration. (**d**) Maximum opening stress along the longer stress distribution and along the notch, specimen PN, material HW, temperature +20 °C, last step of the integration.

It was concluded from the results listed in Table 2 that the final ductile failure process initiated at the center of the C1 specimen and next to the notch in the C04 specimen. Similar results were found for the PN specimen (Figure 7). In this case, the critical spot was either close to the longer axis at the specimen’s centre or next to the notch at the specimen’s central part (see Table 3).

As a result of the observations and computations, each of the critical accumulated effective plastic strains was estimated as a function of the *η* and *L* parameters. Using these values and the least square method, the surfaces of the critical strains were estimated in the *η*, *L*, *ε_eff_pl_cr_* space for all three materials and temperatures. The equation used in the least square method is as follows:

(12) $$\varepsilon_{eff\_pl\_cr}=\omega \exp\left(\beta \eta \right)+\left(a\eta +b\right)L^{2}+c\eta +d\;$$

**Figure 8:**
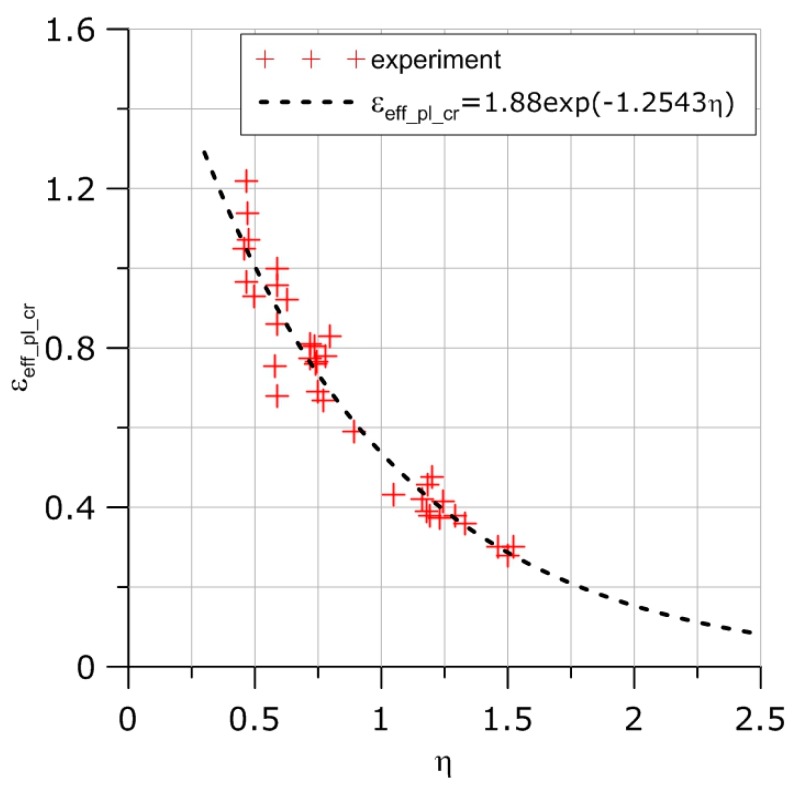
Exponential dependence between the critical strain and the triaxiality factor.

Parameters *ω* and *β* were determined as the coefficients of the exponential function in Equation (12). This function approximates all experimental points for the three materials, three temperatures, and five specimen geometries tested. The experimental points and the trend line are shown in Figure 8. The two parameters were *ω* = 1.88 and *β* = −1.25.

In Table 4, all parameters other than *ω* and *β* in Equation (12) are shown.

**Figure 9:**
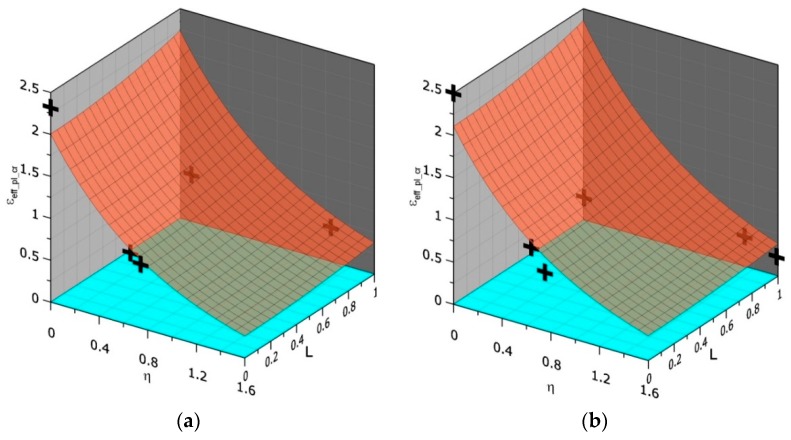
Three-dimensional dependence between *η*, *L*, and *ε_eff_*__*pl*_*cr*_ for (**a**) N material and (**b**) HW material.

The exemplary surfaces for materials N and HW and temperature +20 °C are shown in Figure 9. To draw these surfaces, the values of the coefficients in Equation (12) are as follows: *a* = −0.072, *b* = 0.233, *c* = −0.074, *d* = 0.126 (material N) and *a* = −0.099, *b* = 0.252, *c* = −0.118, *d* = 0.227 (material HW).

## 5. Stress Distribution in Front of the Crack

**Figure 10:**
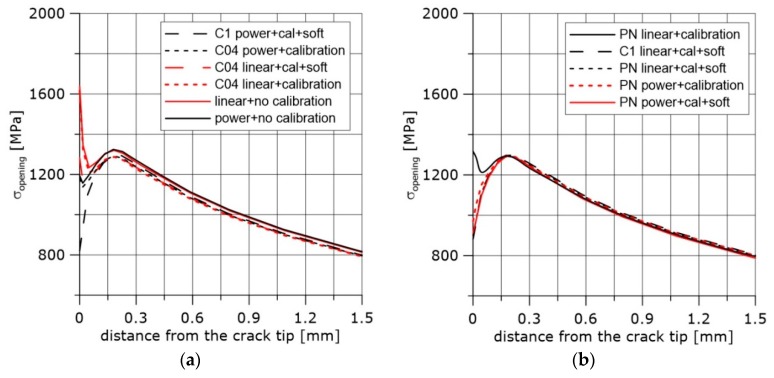
Opening stress distribution computed numerically using stress–strain curves after several calibration procedures. (**a**,**b**) differ by the method of calibration and specimen used.

The numerically computed stress distributions in front of the crack in the SEN(B) specimen depend on the calibration process of the constitutive equations. The exemplary curves are shown in Figure 10 for uncalibrated stress–strain curves and for various calibration procedures.

The details of the specimen geometry and the finite element computations are shown in Section 1.

Note that the calibration that took into account material softening at the last stage of loading led to acceptable results from the physical point of view; the curves went down towards the crack tip after the stress maximum was reached. It was not very important what shape of specimen was used for the calibration, provided the stress triaxiality was high enough. The stress maximum in front of the crack after calibration of the constitutive equation (N material, temperature +20 °C) was lower by 2.6% compared to the results obtained after computation without calibration. The stress maximum was in the range of 1291 to 1297 MPa for the results of computations obtained using calibrated stress–strain curves. The difference between the distances of the stress maximum from the crack tip was within the range of 4 μm. Thus, it was concluded that if the region next to the crack front is not of interest to the researcher, the calibration procedure can be ignored.

## 6. Accumulated Effective Plastic Strain Distributions in Front of the Crack

**Figure 11:**
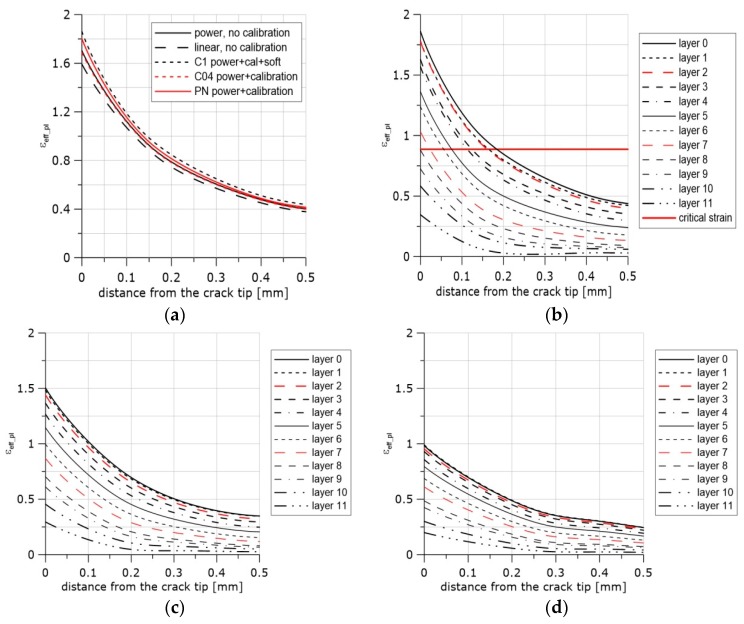
Distributions of the effective plastic strain along the crack front. (**a**) Curves recorded at the final moment of loading after calibration using specimen R1 and the material softening option, material N, temperature +20 °C. (**b**) Curves recorded at the final moment of loading after calibration using specimen R1 and the material softening option, material N, temperature +20 °C. Strain distributions are presented for all layers through the specimen thickness. (**c**) Curves recorded at the 25th/30th step of loading after calibration using specimen R1 and the material softening option, material N, temperature +20 °C. (**d**) Curves recorded at the 20th/30th step of loading after calibration using R1 specimen and the material softening option, material N, temperature +20 °C.

In Figure 11a, selected exemplary curves (5 of 16) of the strain distributions in front of the crack are presented. The difference between the values of the effective plastic strain at the blunted crack tip obtained after computations using uncalibrated stress–strain curves and calibrated curves including the process of material softening was 15%. In Figure 11b–d, the effective plastic strain distributions are presented for all layers through the specimen thickness for three selected steps of integration. These curves may be used together with the results presented in Figure 9 (Equation (12) and Table 4) to estimate the onset and location of the ductile failure mechanism in front of the crack. The first example concerns the SEN(B) specimen made of the N steel tested at +20 °C.

**Figure 12:**
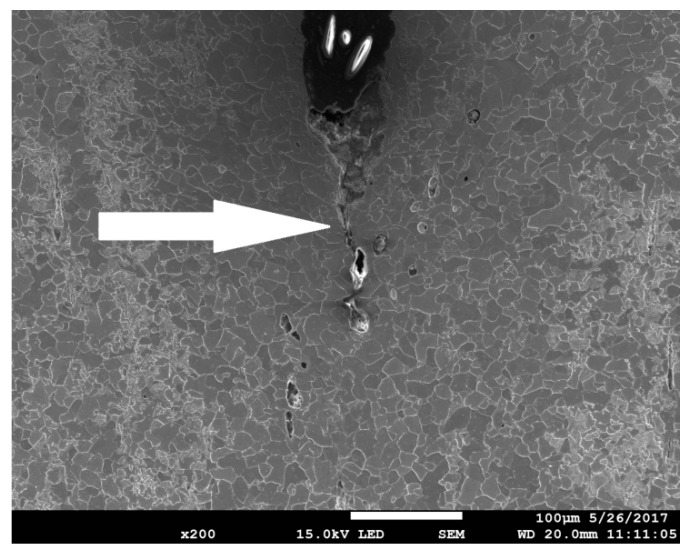
The image of the surface in front of the crack located at the specimen center perpendicular to the crack front, material N, temperature +20 °C. Arrow indicates the crack extension by voids mechanism.

In Figure 12, an image of the middle part of the SEN(B) specimen after unloading is shown. After the test was interrupted, the specimen was unloaded and then cut off along the central surface of the specimen in the perpendicular direction to the crack front. Next, the machined surface was polished and etched. The image of the crack tip and the voids enabled the extension of the crack to be assessed. The crack extension at this location along the crack front due to voids nucleation and coalescence of voids was in the range of 170–190 μm. The coefficients were *η* = 0.67 and *L* = 0.03. According to Equation (12), *ε_eff_pl_cr_* = 0.887. The strain distributions along the crack front at the moment of unloading are shown in Figure 11b. The line denoting the critical strain is also shown. One can easily read from these curves that the crack might grow to a distance of approximately 180 μm, and it is very likely that the crack had also grown until layer 8 along the crack front was reached. One can also presume that the process of nucleation and growth of voids might have started at the middle part of the specimen in front of the crack at the 20th step of loading (maximum number of the loading steps was 30), see Figure 11d.

**Figure 13:**
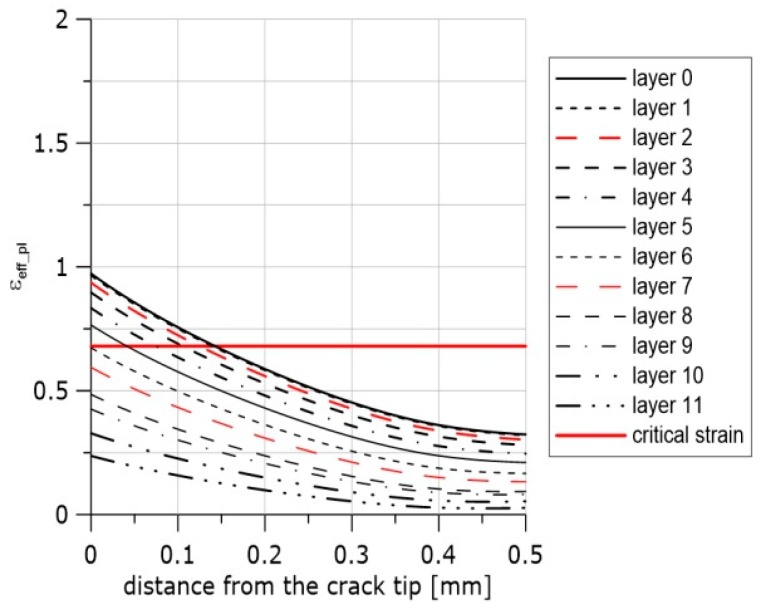
Distributions of the effective plastic strain along the crack front at the final moment of loading after calibration using specimen PR and the material softening option, material NW, temperature −50 °C.

**Figure 14:**
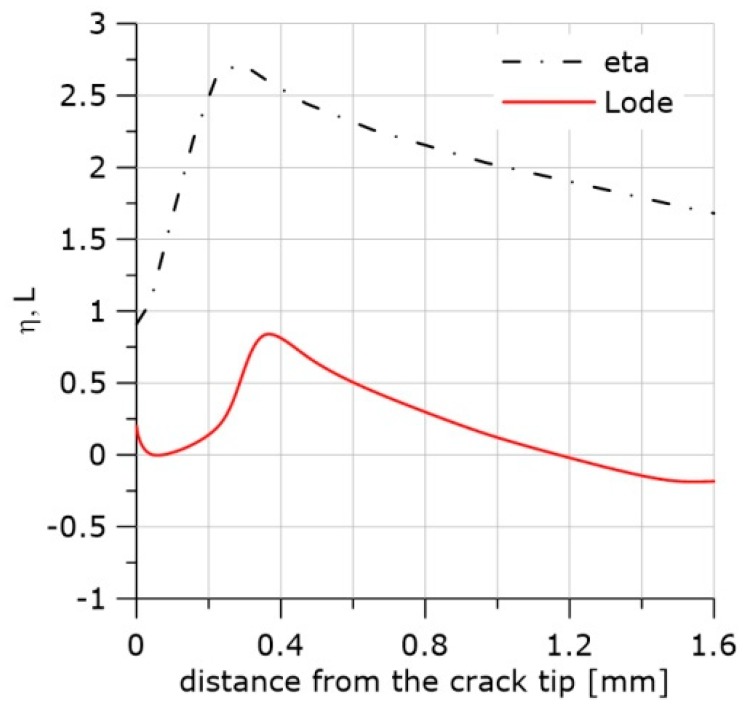
Distributions of the η and Lode functions at the crack center at the moment when the loading was stopped, specimen PR and the material softening option, material NW, temperature −50 °C.

**Figure 15:**
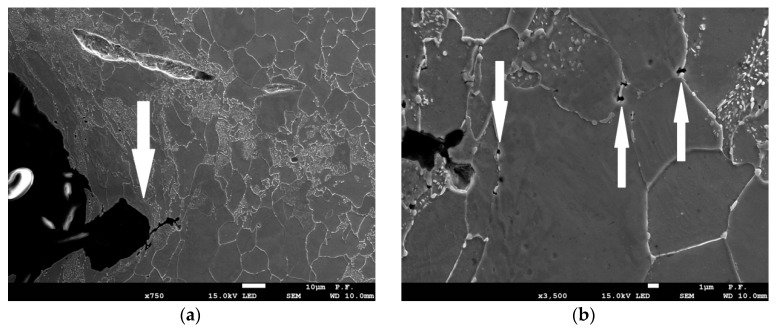
The images of the domain in front of the crack at the moment of unloading. (**a**) The ductile failure mechanism evolves, arrow indicates ductile crack extension, material NW, temperature −50 °C. (**b**) The new voids are visible in front of the growing crack.

Another example is shown in Figure 13, Figure 14 and Figure 15. The SEN(B) specimen was made of NW steel and tested at −50 °C. When the loading was interrupted, the following values were recorded at the crack tip: *η* = 0.81, *L* = 0.22, and *ε_eff_pl_cr_* = 0.68. In this case, the plastic strain distribution in front of the crack suggests that the crack front might have grown to approximately 160 μm. The microscopic image shows that the crack had grown at this plane by 70–80 μm; however, new voids had already grown in front of the growing crack at the distance of 40–50 μm.

**Figure 16:**
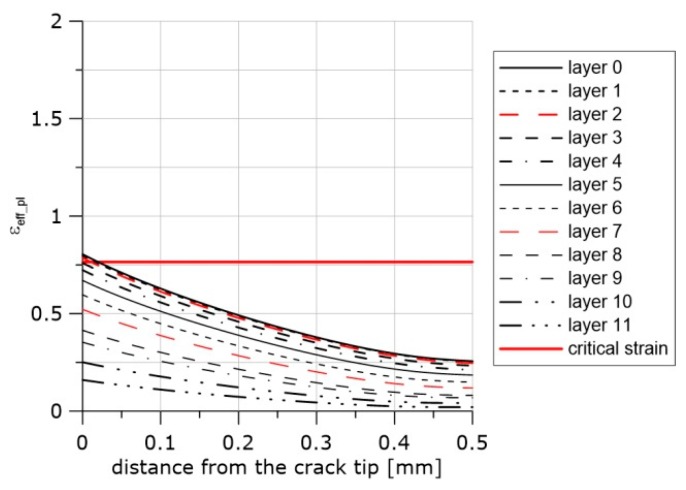
Distributions of the effective plastic strain in front of the crack along the crack front at the final moment of loading after calibration using specimen PR and the material softening option, material HW, temperature +20 °C.

**Figure 17:**
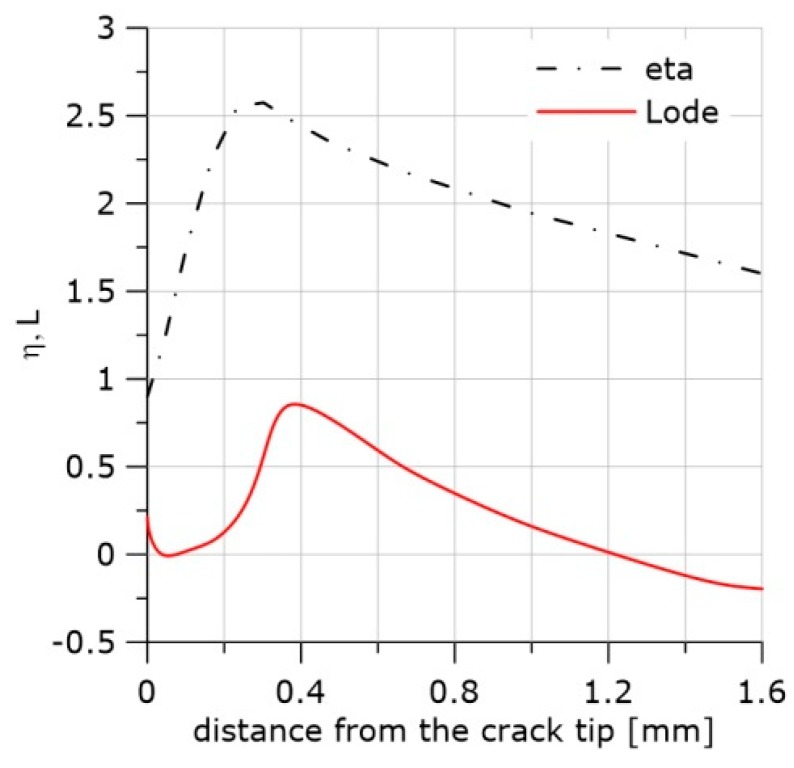
Distributions of the η and Lode functions in front of the crack at the crack centre at the moment when the loading was stopped; specimen PR and the material softening option, material HW, temperature +20 °C.

**Figure 18:**
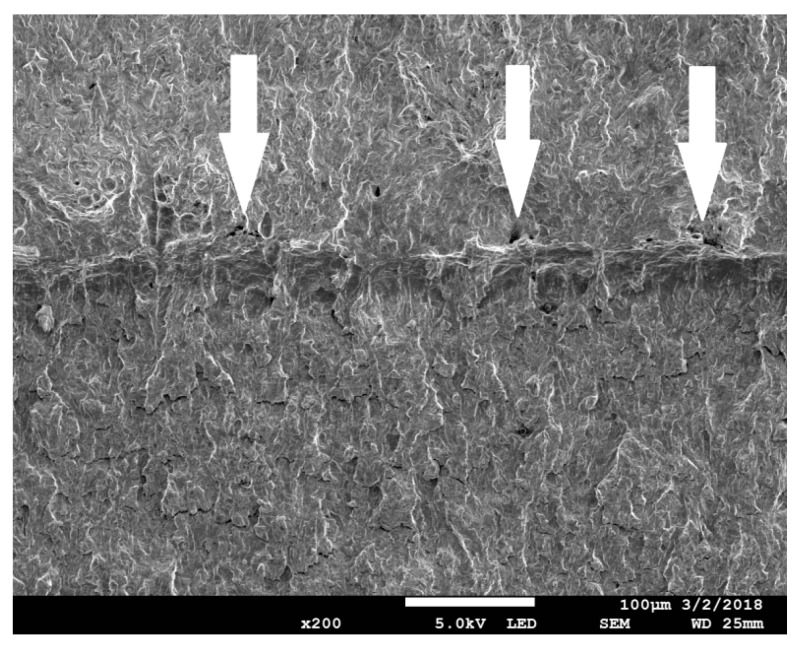
The image of the fracture surface obtained using the scanning microscope. Arrows indicate first voids along the blunted crack tip.

The next example is shown in Figure 16, Figure 17 and Figure 18. The loading of the SEN(B) specimen made of the HW material and tested at +20 °C was interrupted before the onset of crack extension. In the image shown in Figure 18, one may observe in the fracture surface, trace of a blunted crack front and individual voids ahead of the blunted crack front. After the specimen unloading, the fatigue loading was applied until the final failure. At the unloading, the following values were recorded at the crack tip: *η* = 0.88, *L* = 0.22, and *ε_eff_pl_cr_* = 0.76 (see Figure 17 and Equation (12)). The image of the fracture surface and the effective plastic strain distribution in front of the crack at the moment of the specimen unloading indicated that the loading process had been interrupted just before the onset of the crack extension.

## 7. Discussion, Summary and Conclusions

It has been widely accepted since the famous papers by McClintock [19] and Rice and Tracey [18] that the stress triaxiality factor *η* plays an important role in the theoretical analysis of voids growth. Bao and Wierzbicki [1] noticed that the Lode angle (factor) also played an important role in ductile failure prediction and in Reference [2], the authors discussed application of these parameters in various ductile failure criteria. The average values of *η* and Lode angle were used in the process of calibration of discussed criteria. Several authors, listed in the Introduction, have also indicated an important role of both parameters in the evolution of voids and ductile failure. The aim of this research program was to estimate when and where the crack front would start to propagate due to the voids nucleation–growth–coalescence mechanism. It was assumed that this process would initiate at the location where the effective plastic strain reached the critical value, which in turn would depend on the η and Lode factors. As both *η* and Lode factors changed in front of the crack, the experimental program was necessary to compute the function *ε_eff_pl_cr_* = *f(η*,*L)* using the finite element method. Experimental program was performed using five different specimen shapes to generate a wide range of *η* and Lode factors. To localize the critical spot within these specimens, where all quantities were measured in the subsequent integration steps, Equation (11) was postulated. This assumption was necessary as observations of the fracture surface using scanning electron microscopy were not sufficient to draw unique conclusions concerning localization of the critical spot where failure started. To localize the critical spot and critical moment in loaded specimen containing the macroscopic crack, several SEN(B) specimens were machined and loaded. Recorded values of the specimen deflection were used as the boundary values in the finite element analysis. Some specimens had been unloaded before the presumed critical moment was reached, and the fracture extensions were observed using images from scanning electron microscope. Comparison of the microscope images with results obtained in the numerical analysis confirmed the ability of the assumed methodology to estimate the onset of ductile crack growth. The methodology consisted of the following steps:

1. selection of the material;
2. calibration of the constitutive equations, including material softening before the final specimen break;
3. machining of the specimens of at least five different shapes to generate a wide range of *η* and *L* factors;
4. loading of the specimens to failure;
5. localization of the critical spot;
6. computations at the critical spot, by finite element method, the values of *η* and *L* parameters and effective plastic strain;
7. computation of the *ε_eff_pl_cr_* = *f(η*,*L)* function;
8. computation of the mechanical field parameters in front of the crack in the specimen or structural member; and
9. using criterion *ε_eff_pl_* = *ε_eff_pl_cr_* to estimate the onset of ductile crack growth.

The method presented in this paper to estimate the onset of ductile crack extension may be helpful for investigators who are interested in the local analysis of crack growth. Classical procedures to assess the critical moment in ductile materials (e.g., ASTM E1820-18 Standard Test Method for Measurement of Fracture Toughness) do not allow for such estimations, for example, the *J*_IC_ value is measured when the average crack front extension is equal to 0.2 mm.

## Figures and Tables

**Table 1:** The mechanical properties of the materials tested in the research programs.

Materials	Heat Treatment	Microstructure	Temp. (°C)	*E* (GPa)	*R*_eL_ (MPa)	*R*_eH_ (MPa)	*R*_m_ (MPa)
S355JR steel, symbol NW	Normalized and annealed (600 °C, 150 h)	Ferrite containing spheroidal carbide particles	+20	210	382	368	470
			−20	200	376	419	502
			−50	212	390	396	526
S355JR steel, symbol HW	Quenching in oil and annealed (600 °C, 150 h)	Ferrite containing spheroidal carbide particles	+20	197	412	406	511
			−20	191	437	444	555
			−50	210	463	488	581
S355JR steel, symbol N	Normalized at 950 °C	Ferrite–pearlite	+20	197	367	375	496
			−20	202	402	407	526
			−50	220	401	428	553

**Table 2:** Values of the mechanical field parameters at the critical moment along the fracture surface for C04 and C1 specimens, material HW, temperature +20 °C.

Spot Where the Measurements were Performed		*ε_eff_pl_cr_*	*η*	*L*	*σ_max_*	*ε_eff_pl_cr_*·exp*(η)*
R = 0.4	Specimen center	0.24	1.6	0.99	1443	1.19
	Next to the notch	0.93	0.497	0.54	789	**1.53**
R = 1.0	Specimen center	0.36	1.33	0.996	1298	**1.36**
	Next to the notch	0.57	0.42	0.78	619	0.86

**Table 3:** Values of the mechanical field parameters at the critical moment along the PN specimen fracture surface. Material N. Temperature +20 °C.

Spot Where the Measurements were Performed		*ε_eff_pl_cr_*	*η*	*L*	*σ_max_*	*ε_eff_pl_cr_·*exp*(η)*
PN specimen	Central part next to the axis	0.27	1.3	0.033	1278	0.99
	Central part next to the notch	0.92	0.626	0.013	477	**1.16**

**Table 4:** Parameters entering Equation (12) for the three materials and three temperatures tested.

Material	HW	HW	HW	N	N	N	NW	NW	NW
Temp.	−20	−50	20	−20	−50	20	−20	−50	20
a	−0.065	−0.016	−0.099	−0.038	−0.01	−0.07	−0.04	−0.030	−0.12
b	0.121	0.036	0.25	0.113	0.018	0.23	0.11	0.097	0.33
c	−0.046	−0.02	−0.118	−0.07	−0.026	−0.07	−0.087	−0.01	−0.015
d	0.135	0.032	0.227	0.14	0.064	0.126	0.21	0.0024	−0.0027
